# Association between eyeball asymmetry and offset of openings in optic nerve head canal assessed by posterior polar eyeball topography

**DOI:** 10.1038/s41598-024-60716-0

**Published:** 2024-04-30

**Authors:** Kyoung Min Lee, Jung Hyo Rhim, Hyoung Jun Ahn, Martha Kim, Sohee Oh, Sun-Won Park, Seok Hwan Kim

**Affiliations:** 1 Department of Ophthalmology, Seoul National University College of Medicine, Seoul, Korea; 2 Department of Ophthalmology, Seoul National University Boramae Medical Center, 39 Boramae Road, Dongjak-gu, Seoul, 07061 Korea; 3 Department of Radiology, Seoul National University Boramae Medical Center, Seoul, Korea; 4 Department of Mathematical Modeling, Mind Flow Lab, Seoul, Korea; 5 Department of Ophthalmology, Dongguk University Ilsan Hospital, Goyang, Korea; 6 Department of Biostatistics, Seoul National University Boramae Medical Center, Seoul, Korea; 7 Department of Radiology, Seoul National University College of Medicine, Seoul, Korea; 8 Department of Ophthalmology, The One Seoul Eye Clinic, Seoul, Korea

**Keywords:** Anatomy, Medical research

## Abstract

We investigated three-dimensional (3D) eyeball protrusion and its association with the offset between the lamina cribrosa (LC) and Bruch’s membrane opening (BMO). 3D-MRI scans were taken from 93 subjects (186 eyes). An ellipsoid was fitted along the posterior 2/3 contour of each eyeball. Eyeball asymmetry with focal bulging was determined by the existence of an adjacent outward protrusion/reciprocal inward depression pair, and the angular deviation of the outermost protruded point (OPP) was measured from the nasal side of the fovea-BMO axis. The LC/BMO offset was evaluated by measuring the central retinal vascular trunk (CRVT) location from the BMO center: (1) the angular deviation and (2) the offset index as the ratio between the CRVT-BMO center distance and the BMO radius in the same direction. Seventy-nine eyes (42%) were classified as having eyeball asymmetry, which had a more superior LC/BMO offset (*P* < 0.001) and a larger offset index (*P* = 0.002). In those eyes, the angular deviation of the OPP showed a significant correlation with that of the LC/BMO offset (*r* = -0.724, *P* < 0.001), as did protrusion depth with the offset index (*r* = 0.291, *P* = 0.009). The presence of eyeball asymmetry was associated with superior LC/BMO offset (*P* = 0.004) and larger offset index (*P* = 0.009). Superior LC/BMO offset was associated with older age (*P* < 0.001), shorter axial length (*P* < 0.001) and inferior location of OPP (*P* < 0.001). The location and extent of focal bulging were closely associated with those of LC/BMO offset. This indicates that focal bulging during expansion might be associated with diverse directionality of LC/BMO offset.

## Introduction

Visual signals are transferred to the brain via the retinal ganglion cells (RGCs), whose axons exit the eyeball through a gateway: the optic nerve head (ONH). The ONH being a three-dimensional (3D) structure with multiple layers, its morphology is determined by the offset of openings in different layers^1–7^. Offset is incurred as a result of expansion undergone by the eyeball during growth. Since the inner retinal structure of the posterior polar area is relatively unaffected while the outer load-bearing structure expands, their openings, the Bruch’s membrane opening (BMO) and lamina cribrosa (LC), respectively, incur an offset^8–11^, which effects the contrast between infants’ highly uniform ONH morphology and adults’ relative morphologic diversity^12^.

LC/BMO offset, which leads to ONH-morphologic diversity, can be quantified by the central retinal vascular trunk (CRVT)’s deviation from the BMO center, since the CRVT is embedded in the dense connective tissue of the LC and, in most newborns, is located in the central area of the ONH^12^. Actual shift of the CRVT with the appearance of an externally oblique border (EOB) during eyeball expansion has been demonstrated prospectively^8–11^, on which basis, CRVT position could be used as a surrogate for LC position in quantitative evaluation of LC/BMO offset.

The diversity of LC/BMO offset is driven by the diverse modes of eyeball expansion. In a previous study based on three-dimensional MRI (3D-MRI), we found that the offset direction was directly associated with the 3D eyeball shape: prolate expansion induced nasal offset, while oblate expansion induced temporal or superior offset depending on the longest axis of the eyeball^13^. If preferential retinal lengthening in the equatorial region is assumed, prolate expansion would lead to centrifugal shift, and oblate expansion to centripetal shift, of the outer wall opening^13^. Thus, theoretically at least, the LC/BMO offset should be in the nasal direction in the former case and in the temporal direction in the latter. Such hypothesis, however, has not held in cases of asymmetric expansion, where the directionality of LC/BMO offset was from the undergrowth to the overgrowth part^13^. If eyeball asymmetry could be measured quantitatively, we could evaluate the effect of focal bulging on LC/BMO offset more accurately in such cases. Even so, to the best of our knowledge, quantitative evaluations of posterior polar eyeball shape are limited in number. The purpose of the present study, therefore, was to quantify eyeball asymmetry in order to evaluate the association between 3D eyeball shape and LC/BMO offset.

## Results

This study initially involved 96 subjects who had undergone radial scans of the ONH complex and were willing to have 3D-MRI scanning performed. Of these, 4 subjects did not undergo the scanning (3 subjects withdrew their consent and in the case of 1 subject, a metallic foreign body was detected on the MRI scan), and 4 subjects were excluded due to poor MRI image quality. Further, 2 other subjects were excluded due to poor SD-OCT image quality, and 1 due to bifurcation of the CRVT. This resulted in a final sample of 170 eyes of 85 subjects (110 eyes with open-angle glaucoma, 12 eyes with angle-closure glaucoma, 48 eyes without glaucoma). The subjects were aged 55.0 ± 16.2 years, and had a refractive error of -3.97 ± 4.01 diopters, and an axial length of 25.6 ± 1.9 mm; 42 of the subjects were female (49%).

The eyeballs were classified into two groups: group 1 without asymmetry (91 eyes, Supplemental Video 1), and group 2 with asymmetry (79 eyes, Supplemental Video 2) (Table 1). Group 2 had a more superior direction of LC/BMO offset (61.4 ± 55.1° vs. 10.3 ± 74.1°, *P* < 0.001), a larger offset index (0.68 ± 0.28 vs. 0.54 ± 0.29, *P* = 0.002), and a larger RMSE value (0.025 ± 0.010 vs. 0.017 ± 0.005, *P* < 0.001; Table 1). A logistic regression analysis with random effects revealed that superior angular deviation (OR = 1.044, *P* = 0.004) and larger LC/BMO offset (OR = 395.347, *P* = 0.009) were risk factors for eyeball asymmetry (Table 2).

**Table 1:** Demographic data according to three-dimensional eyeball shape.

	Group 1 Without asymmetry (N = 91)	Group 2 With asymmetry (N = 79)	*P*
Age, years	53.1 ± 16.0	57.2 ± 16.3	0.106*
Sex (Male / Female)	49 / 42	37 / 42	0.362^†^
Axial length, mm	25.5 ± 2.1	25.8 ± 1.6	0.365*
IOP, mmHg	15.2 ± 3.1	15.5 ± 5.2	0.692*
BMO area, mm^2^	3.31 ± 2.04	3.23 ± 1.55	0.765*
Angular deviation of LC/BMO offset, *°*	10.3 ± 74.3	61.4 ± 55.1	< 0.001*
Angular deviation of β-zone PPA, *°*	-0.4 ± 68.3	-6.4 ± 73.4	0.647*
Offset index	0.54 ± 0.29	0.68 ± 0.28	0.002*
RMSE of ellipsoid fitting	0.017 ± 0.005	0.025 ± 0.010	< 0.001*
Number of patients with glaucoma	69 (76%)	53 (67%)	0.207^†^

*IOP* Intraocular pressure, *BMO* Bruch’s membrane opening, *LC* Lamina cribrosa, *PPA* Parapapillary atrophy, *RMSE* Root mean square error.

*Comparison performed using independent-*t* test.

^†^Comparison performed using Chi-square test.

**Table 2:** Factors associated with eyeball asymmetry.

	Univariable analysis			Multivariable analysis*		
	OR	95% CI	*P*	OR	95% CI	*P*
Age, years	1.127	(1.026, 1.237)	0.013	0.996	(0.900, 1.103)	0.946
Female (vs. male sex)	2.344	(0.141, 38.937)	0.552			
Axial length, mm	1.503	(0.708, 3.193)	0.289			
IOP, mmHg	1.067	(0.782, 1.457)	0.682			
BMO area, mm^2^	1.044	(0.619, 1.759)	0.872			
**Angular deviation of LC/BMO offset, °**	**1.039**	**(1.013, 1.065)**	**0.003**	**1.044**	**(1.014, 1.076)**	**0.004**
**Offset index**	**84.306**	**(1.985, 3580.172)**	**0.020**	**395.347**	**(4.367, 35788.68)**	**0.009**
Angular deviation of β-zone PPA, *°*	0.997	(0.983, 1.010)	0.613			
Glaucoma diagnosis	0.028	(0.001, 0.818)	0.038	0.165	(0.012, 2.239)	0.176

*OR* Odds ratio, *CI* Confidence interval, *IOP* Intraocular pressure, *BMO* Bruch’s membrane opening, *LC* Lamina cribrosa, *PPA* Parapapillary atrophy.

Statistically significant values (*P* < 0.05) are shown in bold. *Variables with *P* < 0.10 in the univariable analysis were included in the subsequent multivariable analysis.

**Figure 1:**
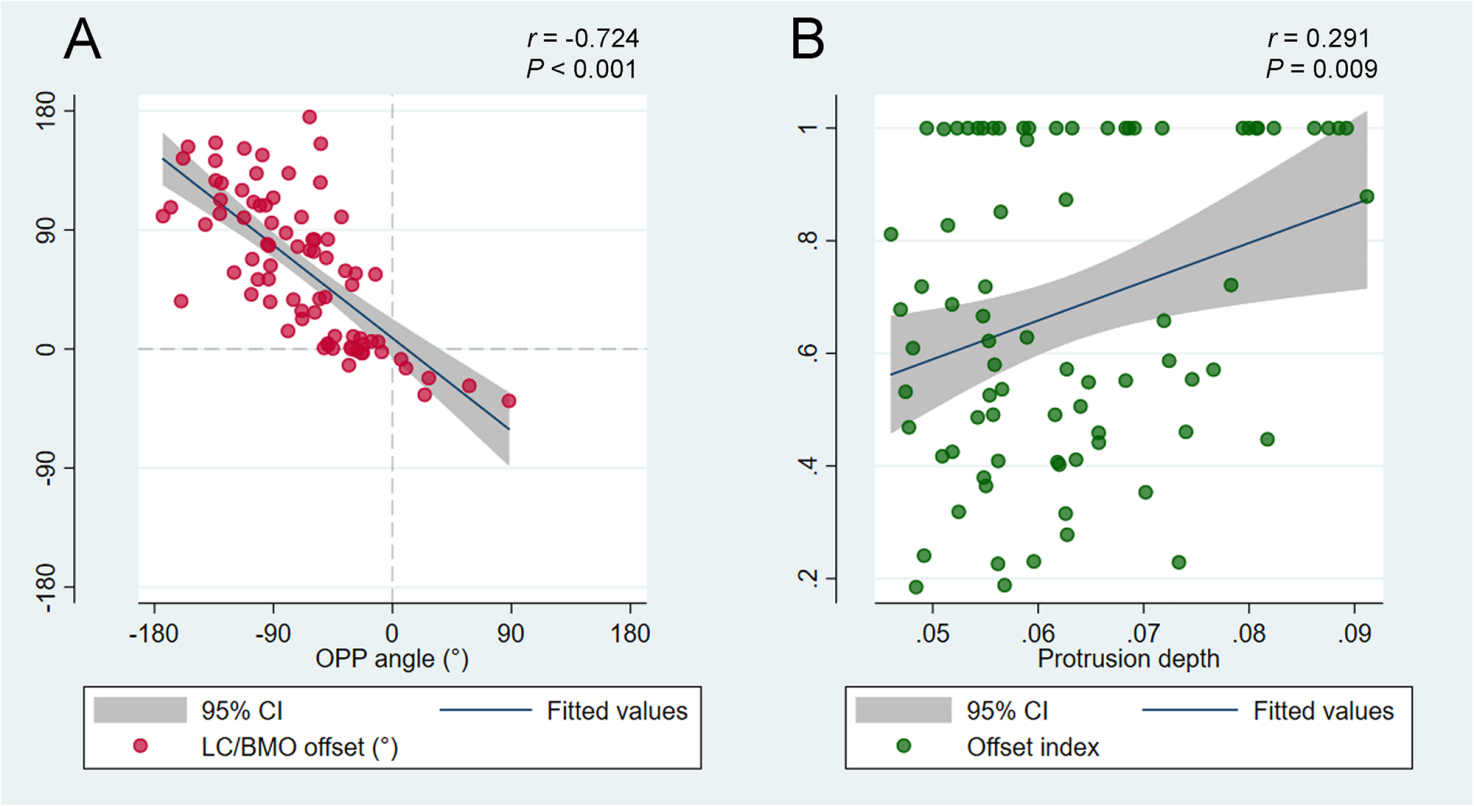
Correlations of angular deviation (**A**) and extent (**B**) between the outermost protruded point (OPP) and lamina cribrosa (LC) / Bruch’s membrane opening (BMO) offset. (**A**) A scatter plot shows the spatial correlation between the LC/BMO offset and the OPP. The LC/BMO offset is in the direction opposite to the OPP location (*r* = − 0.724, *P* < 0.001). (**B**) A scatter plot shows positive correlation between offset index and protrusion depth (*r* = 0.291, *P* = 0.009).

**Figure 2:**
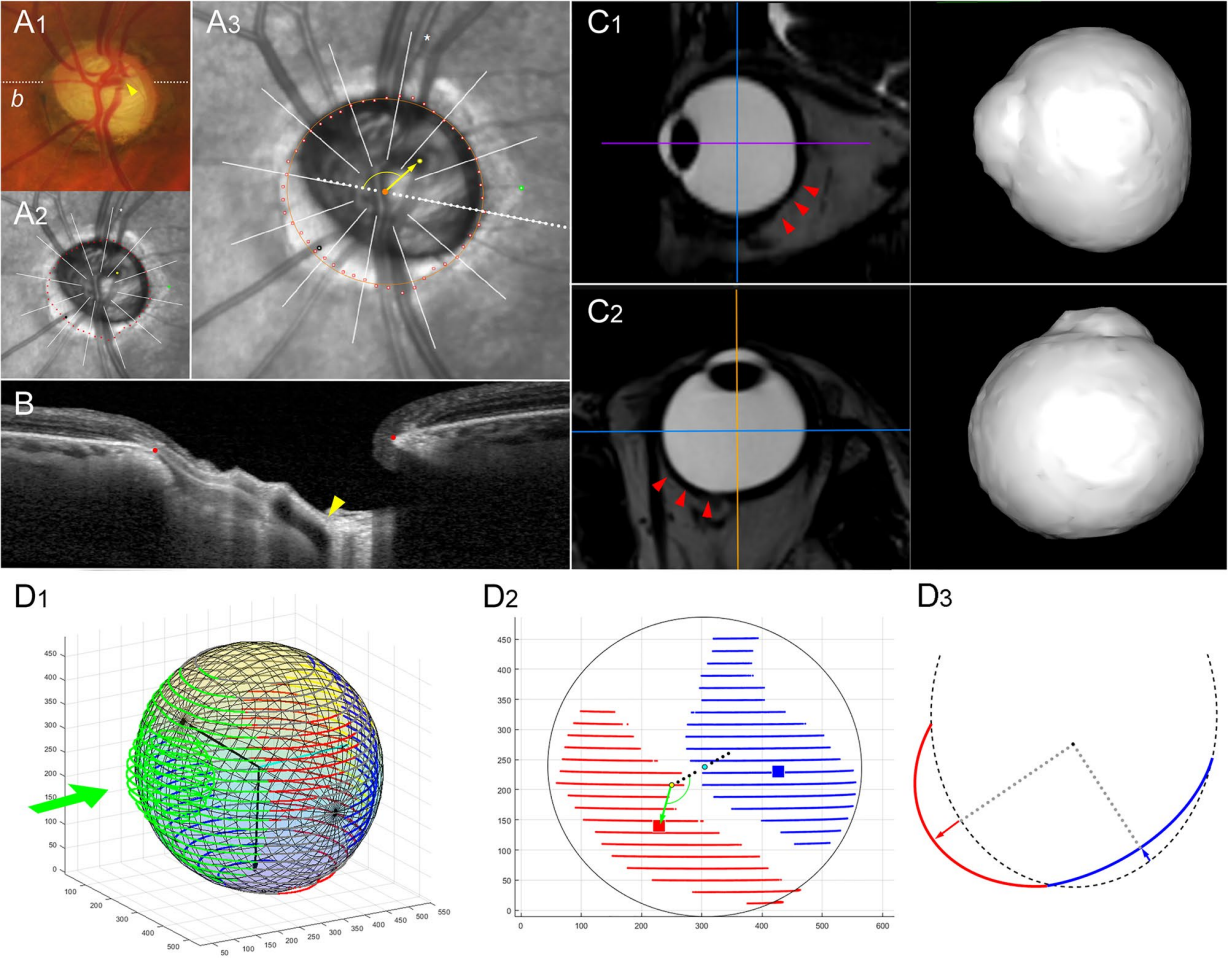
Sample case (left eye) of eyeball asymmetry (group 2). (**A**_**1**_) Disc photograph. The arrowhead indicates the location of the central retinal vascular trunk (CRVT). The dotted white line indicates the locations of the OCT scans. (**A**_**2**_) Infrared image with demarcated Bruch’s membrane opening (BMO) margin (red dots). The CRVT position (yellow dot), the meridian of the longest externally oblique border (EOB, black dot), and the meridian of the maximal width of β-zone parapapillary atrophy (PPA, green dot) are marked on the same infrared image. (**A**_**3**_) Measurement of lamina cribrosa (LC) / Bruch’s membrane opening (BMO) offset. The yellow arrow is drawn to show the direction and extent of CRVT (yellow dot) deviation from the BMO center (orange dot). (**B**) B-scan image. The red dots indicate the BMO margin and the yellow arrowhead indicates the CRVT. (**C**) Sagittal (**C**_**1**_) and transverse (**C**_**2**_) sectional images of three-dimensional (3D) MRI. Please note the asymmetric expansion of the eyeball on the inferior and nasal sides (red arrowheads). (**D**_**1**_) 3D shape evaluated by optimal ellipsoid fitting. (**D**_**2**_) The outermost protruded point (OPP) is on the inferonasal side (green arrow) from the fovea-BMO axis (dotted black line), which is in the direction opposite to the superotemporal LC/BMO offset (**A**_**3**_, yellow arrow). The blue dots indicate the location of fovea, and the yellow dots that of the BMO. The red squares indicate the outermost protruded points, and the blue squares indicate the reciprocal innermost depressed points. (**D**_**3**_) From the best-fitted ellipsoid (black dashed line), the average distance was calculated for the outward protrusion (red line) and inward depression (blue line) as a ratio relative to the radius (gray dotted line) in each direction, respectively, and their summation was defined as the protrusion depth. The entire 3D shape of the eyeball is available in Supplemental Video 2.

**Figure 3:**
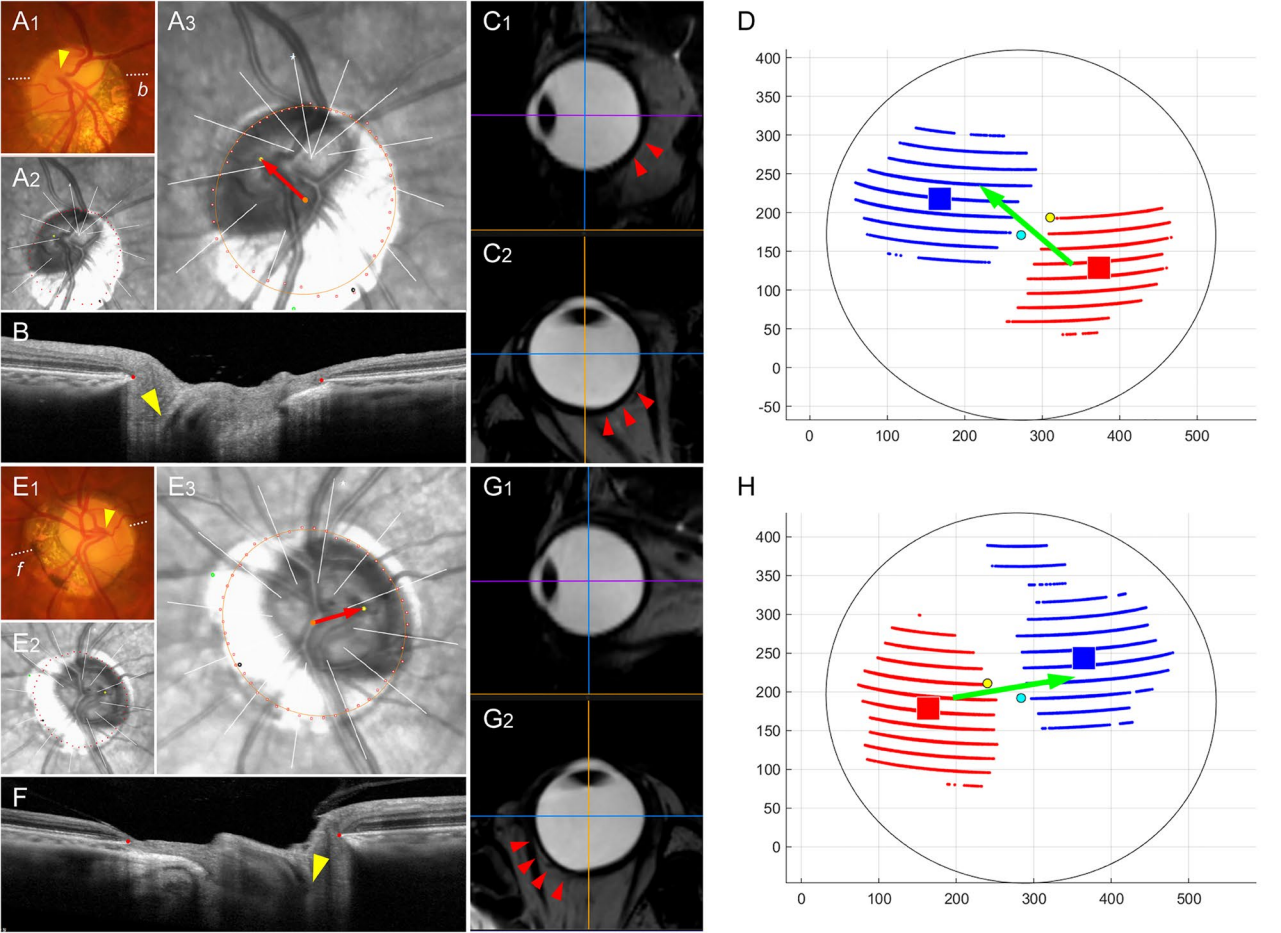
Sample cases of intra-individual difference. (**A**–**D**) Right and (**E**–**H**) left eyes of same subject. (**A**_**1**_, **E**_**1**_) Disc photographs. The arrowheads indicate the locations of the central retinal vascular trunk (CRVT). The dotted white lines indicate the locations of the OCT scans. (**A**_**2**_, **E**_**2**_) Infrared images with demarcated Bruch’s membrane opening (BMO) margin (red dots). The CRVT positions (yellow dot), the meridians of the longest externally oblique border (EOB, black dot), and the meridians of the maximal width of β-zone parapapillary atrophy (PPA, green dot) are marked on the same infrared images. (**A**_**3**_, **E**_**3**_) Measurement of lamina cribrosa (LC) / Bruch’s membrane opening (BMO) offset. The red arrows are drawn to show the direction and extent of CRVT (yellow dots) deviation from the BMO center (orange dots). (**B**,** F**) B-scan images. The red dots indicate the BMO margin and the yellow arrowheads indicate the CRVTs. (**C**,** G**) Sagittal and transverse sectional images of three-dimensional (3D) MRI. Asymmetric expansion is noted by the red arrowheads (**D**,** H**) 3D eyeball shapes are evaluated by optimal ellipsoid fitting. The blue dots indicate the location of the fovea, and the yellow dots that of the BMO. The red squares indicate the outermost protruded points, and the blue squares indicate the reciprocal innermost depressed points. Please note that the LC/BMO offset direction could differ between paired eyes of the same individual, and that it is determined by the 3D shape of the eyeball from the overgrowth to undergrowth directions (green arrows).

In group 2, the OPP was located inferiorly to the fovea-BMO axis (-68.1 ± 51.2°) with a protrusion depth of 0.06 ± 0.01 relative to the radius of the best-fitted ellipsoid. The angular location of OPP showed a negative correlation with direction of LC/BMO offset (*r* = -0.724, *P* < 0.001; Fig. 1A), and protrusion depth showed a positive correlation with offset index (*r* = 0.291, *P* = 0.009; Fig. 1B). In the generalized estimating equation analysis, the superior LC/BMO offset direction was associated with older age (*P* < 0.001), shorter axial length (*P* < 0.001), and inferior location of OPP (*P* < 0.001; Table 3). Posterior polar topography demonstrated the offset direction would be driven by outer-wall shift in the overgrowth-to-undergrowth direction (Fig. 2). The LC/BMO offset direction could differ between paired eyes of the same individual, and was more dependent on the 3D shape of the eyeball (Fig. 3).

**Table 3:** Factors associated with angular deviation of LC/BMO offset.

	Univariable analysis			Multivariable analysis*		
	Coefficient	95% CI	*P*	Coefficient	95% CI	*P*
**Age, years**	**2.459**	**(1.817, 3.101)**	**< 0.001**	**1.217**	**(0.623, 1.811)**	**< 0.001**
Female (vs. male sex)	32.425	(3.665, 61.184)	0.027	− 13.268	(− 29.825, 3.288)	0.116
**Axial length, mm**	**− 21.052**	**(− 27.455, − 14.649)**	**< 0.001**	**− 13.731**	**(− 19.513, − 7.950)**	**< 0.001**
IOP, mmHg	− 0.028	(− 2.867, 2.810)	0.984			
BMO area, mm^2^	− 6.357	(− 13.689, 0.974)	0.089	0.441	(− 4.519, 5.400)	0.862
**Angular deviation of OPP, °**	**− 0.716**	**(− 0.897, − 0.536)**	**< 0.001**	**− 0.377**	**(− 0.545, − 0.210)**	**< 0.001**
Angular deviation of β-zone PPA, *°*	0.997	(0.983, 1.010)	0.613			
Glaucoma diagnosis	12.464	(− 7.895, 32.823)	0.230			

*CI* Confidence interval, *IOP* Intraocular pressure, *LC* Lamina cribrosa, *BMO* Bruch’s membrane opening, *OPP* Outermost protruded point, *PPA* Parapapillary atrophy.

Statistically significant values (*P* < 0.05) are shown in bold. *Variables with *P* < 0.10 in the univariable analysis were included in the subsequent multivariable analysis.

## Discussion

In this study, we developed a new method for 3D evaluation of posterior eyeball shape and assessed its association with LC/BMO offset. For this purpose, we fitted an optimal ellipsoid along the contours of the eyeball, which left a dominant singular protrusion in 46% of eyes. In the eyes with dominant singular protrusion, the direction and extent of LC/BMO offset correlated with the direction and extent of the posterior eyeball protrusion from the best-fitted ellipsoid. This confirms that the offset between the retinal and scleral layers is determined by the relative shift of the outer-wall with its protrusion during eyeball expansion. To the best of our knowledge, this is the first study to evaluate the effect of posterior protrusion on LC/BMO offset.

LC/BMO offset reflects the scleral/retinal layers misalignment^1–4^, incurred during eyeball expansion in the growth period^8–11^. The CRVT position is used as a surrogate of the LC position, since it is embedded in the dense connective tissue composed of multiple collagen sheets^14^. The CRVT is located centrally in newborns^12,15^, and its actual shifting with eyeball expansion has been documented^8–11^. In a previous 3D MRI study, we showed that LC/BMO offset direction was associated with 3D eyeball shape: prolate-spherical with nasal offset, temporally-oblate-spherical with temporal offset, and vertically-oblate-spherical with superior offset^13^. This relationship could be explained by the disproportionate growth of the retina relative to that of the sclera in the corresponding region^13^. In certain cases, however, eyeball shapes were not classifiable simply as prolate or oblate spheres, since they had marked asymmetry; in those cases, the LC/BMO offset direction had been driven by outer-wall shift in the overgrowth-to-undergrowth direction^13^. In this study, we developed a new method for measuring the direction and extent of eyeball asymmetry and correlating them with those of LC/BMO offset. In the results, the direction and extent of eyeball asymmetry correlated well with the direction and extent of LC/BMO offset.

ONH morphology is known to be associated with 3D eyeball shape. Kim et al. evaluated the posterior polar shape by finding the deepest point using swept-source OCT^16–18^. Although symmetric elongation had resulted in the deepest point being near the fovea, asymmetric growth resulted in the deepest point being elsewhere^16^. We speculated that the deepest point being other than near the fovea would correspond to the OPP in cases with asymmetric bulging. Asymmetric expansion of the eyeball, which is reflected by the deepest point or the OPP, might aggravate the offset between the retinal and scleral openings, which had been induced by the relative preservation of the posterior polar retina in the direction of the outer-wall overgrowth.

During the expansion period, the eyeball shape can transform from its spherical shape into diffuse expansion, prolate/oblate spheres, or asymmetric bulging^19^, all of which have been demonstrated in 3D-MRI studies^13,20^. Shinohara et al. showed that patients with tilted disc syndrome have an outward protrusion of the inferior globe^21^. In a previous 3D-MRI study, we observed a similar protrusion in the direction opposite to the LC/BMO offset in eyes with asymmetric bulging^13^. Inferonasal scleral overgrowth may mimic retinal vessels’ situs inversus resulting from a short fovea-disc distance^22^, given a fovea-proximate superotemporal location of CRVT emergence. Therefore, we speculated that tilted disc syndrome could be partially explained by the LC/BMO offset induced by asymmetric bulging, and in fact, that this might be the reason for its exceptionally high prevalence as a congenital optic disc anomaly^23^. In this study, patients with such asymmetric eyeball shapes (group 2) were of older age and had shorter axial length relative to patients with axial myopia without asymmetry. We speculated this was the reason for the association of superior LC/BMO offset with older age and shorter axial length.

Posterior polar eyeball shape has clinical implications through its effect on LC/BMO offset. As for RGCs, their cell bodies are anchored to the retinal plane above the BMO while their axons have to pass through the LC. Therefore, LC/BMO offset would result in more oblique paths through which RGCs must exit the ONH to form synapses with the brain. Moreover, LC/BMO offset is reported to be associated with more focal LC defects in the opposite direction^9,24^. The offset has been found to be larger in glaucomatous eyes than in fellow eyes in unilateral glaucoma^25^. Moreover, glaucomatous optic neuropathy occurrs more frequently^24^ and earlier in the direction opposite to the offset^26,27^. In this paper, we suggest a new method for evaluation of asymmetric bulging that directly induces LC/BMO offset from the overgrowth part to the undergrowth part.

This study has several limitations. First, the LC/BMO offset signifies the relative relationship between the LC and BMO, and thus, growth-related BMO change should be accounted for, especially in highly myopic eyes, before assessing LC deviation from the BMO. Second, our method is limited by the resolution of 3D-MRI. To fit an optimal ellipsoid, data on more than half of the eyeball contour was required, for which reason, we had to use 3D-MRI data; 3D-MRI data, however, has a limitation in terms of precision locating of the fovea and the BMO. Optimal ellipsoid fitting itself is not restricted to 3D-MRI data, and might be improved for use with swept-source OCT, which offers better resolution, in the future. Third, all of the participants were South Korean, though ONH and eyeball morphologies may differ by ethnicity^28^. Further research will be required to validate the current findings for different populations. Fourth, both glaucoma patients and healthy subjects were included, thereby potentially incurring a confounding effect of LC remodeling on the CRVT position in glaucomatous eyes^29^. Glaucomatous ONH change has been reported *not* to affect the CRVT position in the LC^26,30^, and our subgroup analysis using glaucomatous eyes, moreover, showed very similar results (Supplemental Tables 1 and 2). Nevertheless, we could not completely exclude the influence of glaucomatous change on the CRVT position, and this should be acknowledged as a limitation. Fifth and finally, our method considers only the protrusion of the vitreous-retinal interface, not the individual protrusions of each of the layers. Since the LC/BMO offset reflects the differences of all of the layers during eyeball expansion, the posterior polar shapes of each of those layers would be informative. Further study is required to validate this speculation.

In conclusion, eyeball expansion during growth may result in asymmetric bulging at the posterior pole, which was measured by optimal ellipsoid fitting in the present study. The location and extent of bulging from the best-fitted ellipsoid were closely associated with the direction and extent of the LC/BMO offset. Posterior polar evaluation with ellipsoid fitting would be helpful to determinations of the effect of eyeball expansion on ONH anatomy and glaucoma.

## Methods

### Study participants

This investigation was based on subjects who had been enrolled in the Boramae Glaucoma Imaging Study (BGIS), an ongoing prospective study at Seoul National University Boramae Medical Center (Seoul, Korea)^7,13,24–26^. Between June 2019 and February 2022, volunteers who had earlier visited our institution due to a diagnosis or suspicion of glaucoma and expressed a desire for 3D-MRI scans were recruited, and their ONH anatomic features were registered^13^. Written informed consent to participate was obtained from all. The study protocol was approved by the Seoul National University Boramae Medical Center Institutional Review Board and conformed to the tenets of the Declaration of Helsinki.

Subjects who were enrolled in the BGIS underwent a full ophthalmologic examination that included best-corrected visual acuity (BCVA) assessment, refraction, slit-lamp biomicroscopy, Goldmann applanation tonometry, gonioscopy, dilated funduscopic examination, keratometry (RKT-7700; Nidek, Hiroshi, Japan), axial length measurement (IOLMaster version 5; Carl Zeiss Meditec, Dublin, CA, USA), disc photography along with red-free fundus photography (TRC-NW8; Topcon, Tokyo, Japan), and spectral-domain (SD) OCT (Spectralis OCT, Heidelberg Engineering, Heidelberg, Germany)^7,13,24–26^. During acquisition of the SD-OCT images, the subjects were asked to fixate on the target, and images were acquired with the forehead and chin stabilized by the headrest. Extra care was taken to confirm that the forehead and chin were correctly positioned and did not move. Glaucomatous optic nerve damage was defined by rim thinning, notching, and the presence of retinal nerve fiber layer defects, and was evaluated by two glaucoma specialists (KML and SHK). Glaucomatous visual field defect was defined as (1) outside normal limits on glaucoma hemifield test; or (2) 3 abnormal points with a *P* value less than 5% probability of being normal and 1 with a *P* value less than 1% by pattern deviation; or (3) pattern standard deviation less than 5%, as confirmed on 2 consecutive reliable tests (fixation loss rate ≤ 20%, and false-positive and false-negative error rates ≤ 25%). Glaucoma was defined as glaucomatous optic nerve damage with associated visual field defects and was classified into open-angle glaucoma and angle-closure glaucoma according to the irido-corneal angle status as assessed by gonioscopy.

The inclusion criterion was the willingness of subjects to undergo 3D-MRI scans for delineation of eyeball shape. The exclusion criteria were BCVA < 20/40, poor-quality imaging (i.e., quality score < 15) of any section on enhanced depth imaging (EDI) SD-OCT radial scans; CRVT position located within the BMO but impossible to determine clearly due to vessel bifurcation, and any MRI contraindication (e.g., MRI-unacceptable aneurysm clip, pacemaker, any metallic foreign body). Both eyes were used in the analysis.

### Three-dimensional (3D) MRI of eyeball

The 3D shape of the eyeball was measured as an area of high signal index on T2-weighted 3D-MRI images^13^. The MRI examinations were performed at 3.0 T using a Philips Achieva (Philips Healthcare, Best, The Netherlands). The participants were instructed to keep both eyes closed with minimal movement during the scanning. Scanning sequences (repetition time = 2500 ms; echo time = 248 ms; flip angle = 90°; field of view = 256 $\times$ 256 $\times$ 188 mm) were performed with maximum water-fat shift^15^. The image resolution was 1 $\times$ 1 $\times$ 1 mm. Volume rendering of the images was performed using commercially available software (OsiriX MD; FDA cleared, Pixmeo, Bernex, Switzerland).

### Optimal ellipsoid fitting

**Figure 4:**
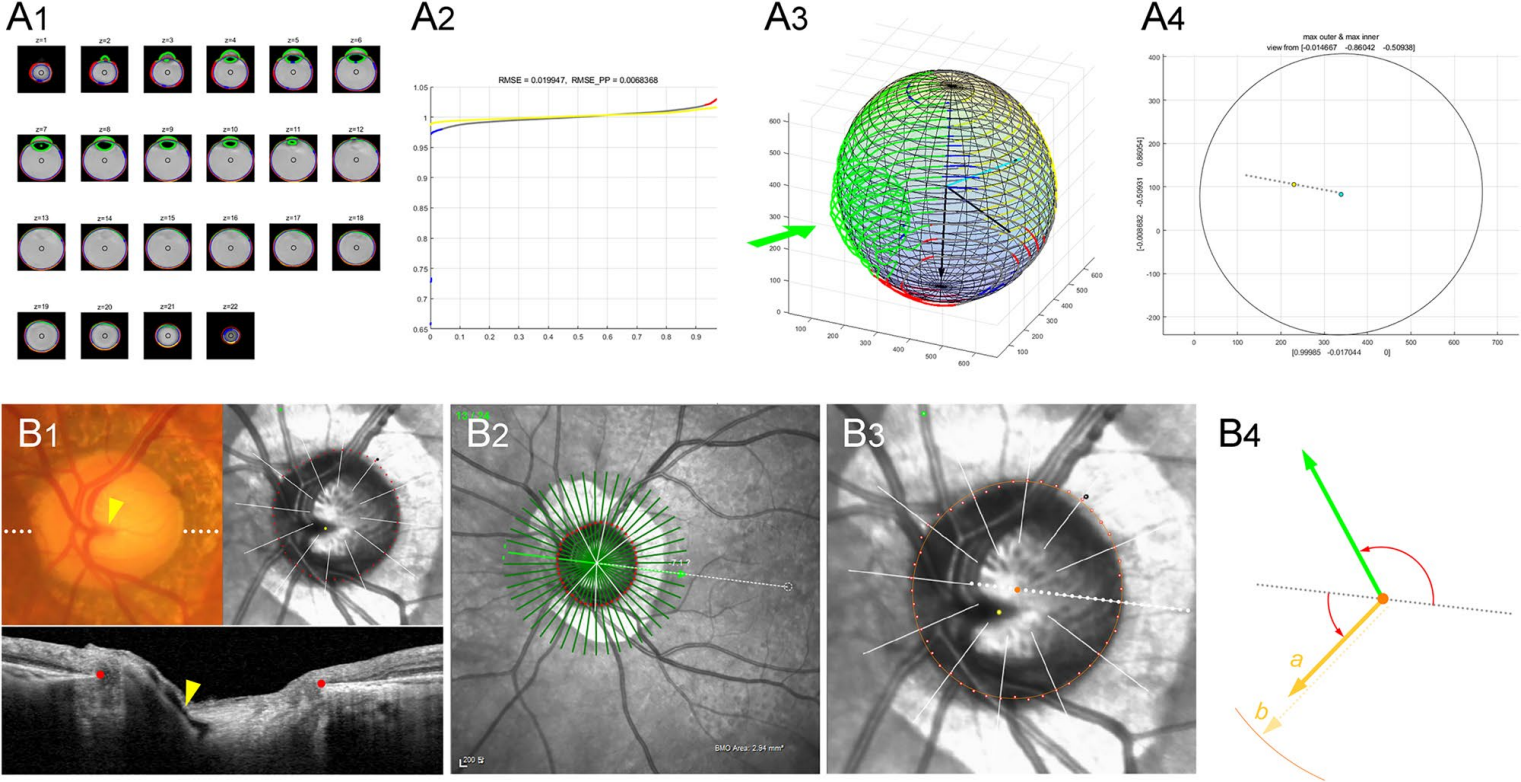
Measurement of posterior polar protrusion (**A**) and offset between lamina cribrosa (LC) and Bruch’s membrane opening (BMO) in optic nerve head canal (**B**). (**A**_**1**_) The eyeball contour is delineated from the stacks of MRI scans using the Canny edge detector. After excluding the anterior 1/3 (green lines), the Cartesian coordinates of the contour are obtained along the posterior 2/3 of the eyeball. Then, an ellipsoid is fitted to minimize the cost function, which is the square sum of all of the distances. (**A**_**2**_) The goodness of fit was evaluated by the root mean square error (RMSE) for every eyeball. (**A**_**3**_) From the best-fitted ellipsoid, the dots outside the ellipsoid (red dots) are classified as outward protrusion, and the dots inside the ellipsoid (blue dots) as inward depression. (**A**_**4**_) For measurement, every point in the x–y–z space is projected onto the posterior polar plane, which is perpendicular to the visual axis. The angular deviation is measured from the line connecting the fovea (blue dot) and BMO (yellow dot): the fovea-BMO axis (gray dotted line). In this eyeball, the ellipsoid fitting leaves no outward protrusion or inward depression. The entire 3D shape of the eyeball is available in Supplemental Video 1. (**B**_**1**_) Disc photograph and OCT image. The LC/BMO offset is measured by central retinal vascular trunk (CRVT) deviation from the BMO center. The white dotted line indicates the location of the OCT scan, as targeted to the CRVT (yellow arrowheads). The B-scan OCT image clearly shows the emergence of the CRVT. The red dots indicate the BMO margin. (**B**_**2**_) Infrared image obtained by OCT. The fovea-BMO axis is demarcated (white dotted line). (**B**_**3**_) Optimal ellipse (orange circle) is fitted along the BMO to obtain BMO center (orange dot). The CRVT position (yellow dot), the meridian of the longest externally oblique border (EOB, black dot), and the meridian of the maximal width of β-zone parapapillary atrophy (PPA, green dot) are marked on the same infrared image. (**B**_**4**_) From the BMO center (orange dot), the angular deviation of CRVT (yellow arrow) is measured clockwise, with the nasal horizontal midline as 0°. A positive value indicates the superior location and a negative value, the inferior location. Likewise, the meridian of the maximal width of β-zone PPA (green arrow) is measured clockwise, with the temporal horizontal midline as 0°. A positive value indicates the superior location and a negative value, the inferior location. From the BMO center, the distances are measured to the CRVT (*a*) and to the BMO margin in the same direction (*b*). The ratio of these distances is defined as the “offset index” (*a*/*b*), which is used to measure the extent of LC/BMO offset.

Using the OsiriX software, the eyeball orientation was aligned along the *z*-axis, and the reference points were marked at the centers of the lens and the ONH, which were projected on the foveal plane to the fovea and BMO, respectively. Then, the stacks of MRI scans were read by our customized codes. First, the eyeball contour was delineated using the Canny edge detector: the gray-scale images of the T2-weighted MRI scans were converted to the linear scale (0 ~ 256), and the edge was traced using the intensity gradient across borders between adjacent pixels. Then, an ellipsoid was fitted along the posterior 2/3 of the eyeball, and the Cartesian coordinates of the contour were obtained in the *x*–*y*–*z* space (Fig. 4A1). An ellipsoid was defined as a second-order polynomial in the *x*–*y*–z space: $a{x}^{2}+b{y}^{2}+c{z}^{2}+dx+ey+fz+gxy+hyz+izx+j=0$. Based on the distances between the ellipsoid and the given set of dots, we defined the cost function, which is the squared sum of all of the distances. The optimal ellipsoid, which is to say, that which minimizes the cost function, was drawn using the Nelder-Mead method (Appendix). The goodness of fit was evaluated by the root mean square error (RMSE) for every eyeball (Fig. 4A2).

From the best-fitted ellipsoid, outward protrusion or inward depression was determined according to whether the dots were outside or inside, respectively (Fig. 4A3). Eyeball asymmetry was defined as dominant outward protrusion existing singularly with an immediately adjacent reciprocal inward depression occupying more than half the area of the posterior pole. Each eyeball was classified into group 1 (eyeball without asymmetry) or group 2 (eyeball with asymmetry). In group 2, the outermost protruded point (OPP) was projected onto the posterior polar plane, which is perpendicular to the visual axis, and its angular deviation was measured from the nasal side of the fovea-BMO axis, a positive value indicating the superior location (Fig. 4A4). The average distance from the best-fitted ellipsoid was calculated for the outward protrusion and the inward depression as a ratio relative to the radius of the best-fitted ellipsoid in each direction, respectively, and their summation was defined as the protrusion depth (Fig. 2D3).

### Assessment of deep-ONH complex

The peripapillary area was imaged by SD-OCT. Prior to performing the scanning, the corneal curvature of each eye was entered into the SD-OCT system (Spectralis, Heidelberg Engineering) so as to compensate for potential magnification error. The deep-ONH complex was imaged using the EDI technique. The BMO was demarcated using the Glaucoma Module Premium Edition of the Spectralis machine. With 24 high-resolution radial scan images of the ONH, 15° apart from each other, and each averaged from 24 individual B-scans, SD-OCT automatically detected the margin of the BMO. Every detected BMO margin was reviewed by one of the authors (KML), and errors were corrected manually. Based on the edited BMO margin, the Spectralis machine calculated the area and center of the BMO^7^.

The LC/BMO offset was measured as the deviation of the CRVT position from the BMO center, as described previously^7^. First, the emergence of the CRVT (artery) was demarcated on fundoscopic infrared images and color-disc photography; then, it was confirmed by cross-sectional SD-OCT imaging in all cases (Fig. 4B1). In cases with an invisible CRVT on infrared fundus photographs and B-scan EDI SD-OCT images, OCT angiography (Spectralis) was used to determine its presence within the BMO. The CRVT deviation from the BMO center was defined in two aspects: (1) its angular deviation, and (2) extent. The angle was measured based on the right-eye orientation, with the nasal fovea-BMO axis as 0° (a positive value indicating a CRVT located superiorly, and a negative value, inferiorly). To evaluate the extent of offset, the distance of the CRVT from the center of the BMO (*a*) was divided by the distance of the BMO margin from the center of the BMO in that direction (*b*) and defined as the “offset index” (Fig. 4B, a/*b*). In cases of CRVT invisibility due to being located outside the BMO, the offset index was defined as 1.0, and the angular deviation of LC/BMO offset was defined as that in the direction opposite to the longest EOB^13^ (Supplemental Fig. 1).

The BMO center was found using our codes that fit the best ellipse for a given set of dots^7^. The CRVT location, the longest EOB, and the angular location of the maximal width of β-zone parapapillary atrophy (PPA) were marked on the same infrared image. Their pixel values were read by our customized software to calculate all of the parameters automatically^7^ (Supplemental Fig. 1).

### Data analysis

Group comparisons were performed using the independent *t*-test for the continuous variables and chi-square test for the categorical variables. Factors associated with asymmetric bulging were evaluated by logistic regression analysis with random effects to account for paired-eye correlations from the same participants. For the group with asymmetric bulging, regression analysis was used to determine the factors affecting the LC/BMO offset direction. The generalized estimating equation regression model was applied to account for paired-eye correlations from the same participant. Univariable and multivariable regression analysis were used to determine the factors; parameters with a *P* value less than 0.10 in the univariable analysis were included in the subsequent multivariable analysis. Statistical analyses were performed with commercially available software (Stata version 16.0; StataCorp, College Station, TX, USA) and R statistical packages version 3.4.3 (available at http://www.r-project.org; accessed December 5, 2017). The data herein are presented as the mean ± standard deviation except where stated otherwise. The cutoff for statistical significance was set at *P* < 0.05.

### Supplementary Information

Supplementary Information 1.

Supplementary Video 1.

Supplementary Video 2.

## Data Availability

The datasets used and/or analysed during the current study available from the corresponding author on reasonable request.
