# Supplementary material for: Association between eyeball asymmetry and offset of openings in optic nerve head canal assessed by posterior polar eyeball topography
Source: Sci Rep. 2024 Apr 30;14:9952. doi: 10.1038/s41598-024-60716-0 (PMC11061147; doi:10.1038/s41598-024-60716-0)
Supplement: Supplementary file 1 — Supplementary Information 1. [file 41598_2024_60716_MOESM1_ESM.docx]

**Appendix.**

Let us represent a general ellipsoid by an implicit second order polynomial:

$ax^{2}+by^{2}+cz^{2}+dx+ey+fz+gxy+hyz+izx+j=0$.

We want to find the initial ellipsoid equation $\left( \hat{a},\hat{b},\hat{c},\hat{d},\hat{e},\hat{f},\hat{g},\hat{h},\hat{i},\hat{j} \right)$ that fits $\left( x_{1},y_{1},z_{1} \right),\cdots, \left( x_{n},y_{n}, z_{n} \right)$ by

$$\left( \hat{a},\hat{b},\hat{c},\hat{d},\hat{e},\hat{f},\hat{g},\hat{h},\hat{i},\hat{j} \right)=argmin\sum_{k=1}^{n} \left( ax^{2}+by^{2}+cz^{2}+dx+ey+fz+gxy+hyz+izx+j \right)^{2}$$

satisfying $(a^{2}+b^{2}+c^{2}+d^{2}+e^{2}+f^{2}+g^{2}+h^{2}+i^{2}+j^{2}=1)$.

This equation can be solved by linear algebra. We note that the equation above is different from the rigorous definition of the cost function, since squared value of the implicit second-order polynomial is not equal to the squared value of the distance between the given ellipsoid and point $\left( x_{k},y_{k},z_{k} \right)$.

Then, our goal is to find the best ellipsoid $M$ that minimizes

$$\sum_{k=1}^{n} d\left( M,\left( x_{k},y_{k},z_{k} \right) \right)^{2}$$

where $d\left( M,\left( x_{k},y_{k},z_{k} \right) \right)$ is the distance between ellipsoid $M$ and point $\left( x_{k},y_{k},z_{k} \right)$.

The mathematical definition is as follows:

$d\left( M,\left( x_{k},y_{k},z_{k} \right) \right)= \underset{\left( x,y,z \right)\in M}{MIN} \sqrt{\left( x-x_{k} \right)^{2}+\left( y-y_{k} \right)^{2}+\left( z-z_{k} \right)^{2}}$.

This optimization problem is solved numerically (by the Nelder-Mead method).


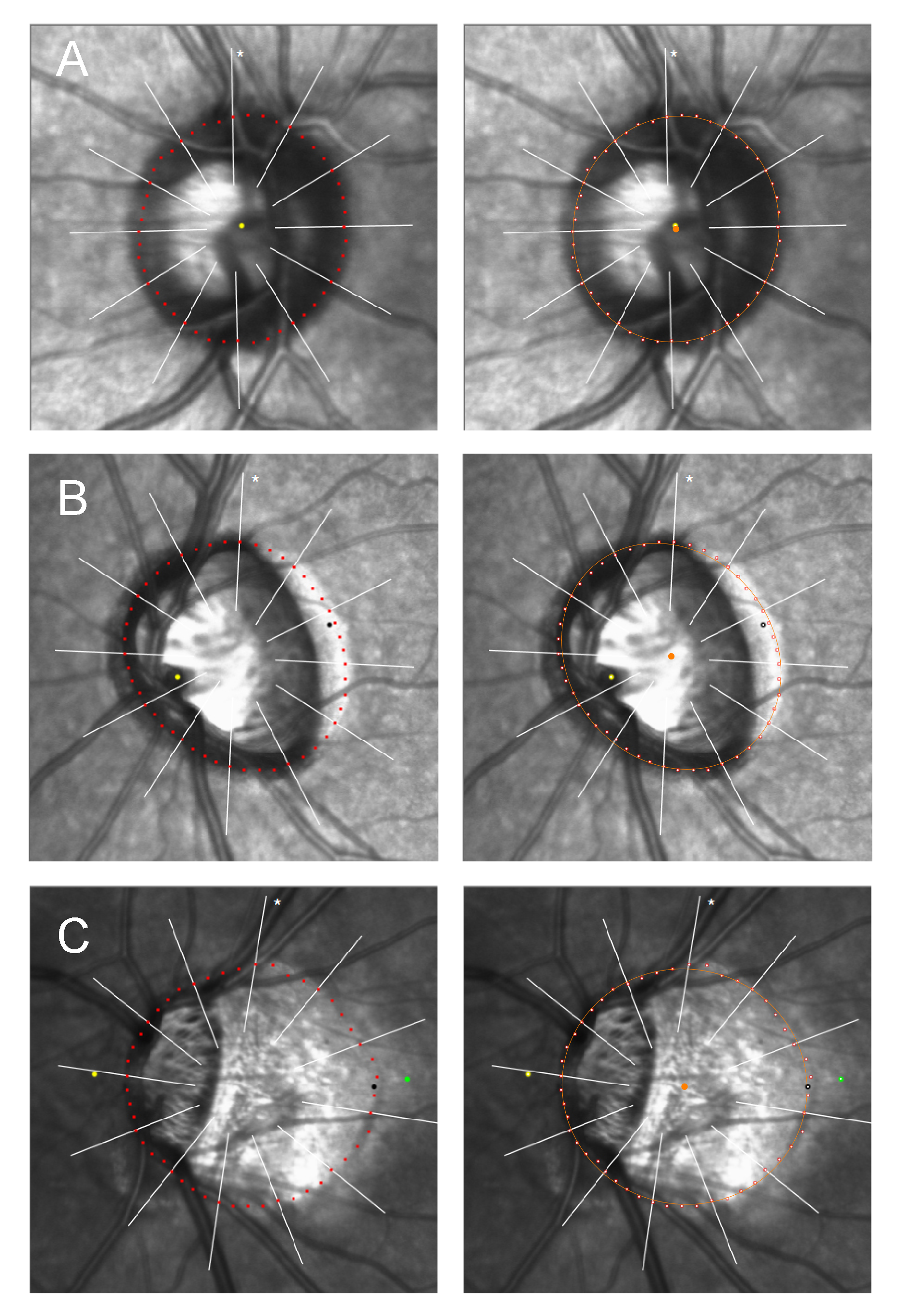


**Supplemental Figure 1.** Diverse extents of offset between lamina cribrosa (LC) and Bruch’s membrane opening (BMO). The red dots indicate the BMO margin, and the yellow dots the central retinal vascular trunk (CRVT). The optimal ellipse (orange circle) is fitted along the BMO to obtain the BMO center (orange dot). The offset index is 0.03 (**A**), 0.61 (**B**) and 1.0 (**C**).

**Supplemental Video 1.** Eyeball without asymmetry (Group 1, Figure 4)

**Supplemental Video 2.** Eyeball with asymmetry (Group 2, Figure 2)

**Supplemental Table 1.** Factors associated with eyeball asymmetry in glaucomatous eyes

|  | Univariable analysis | | |  | Multivariable analysis^*^ | | |
| --- | --- | --- | --- | --- | --- | --- | --- |
|  | OR | 95% CI | *P* |  | OR | 95% CI | *P* |
| Age*, years* | 1.094 | (0.967, 1.238) | 0.155 |  | 0.902 | (0.766, 1.062) | 0.216 |
| Female (vs. male sex) | 0.877 | (0.011, 71.823) | 0.953 |  |  |  |  |
| Axial length, *mm* | 0.916 | (0.380, 2.205) | 0.844 |  |  |  |  |
| IOP, *mmHg* | 1.281 | (0.867, 1.894) | 0.214 |  |  |  |  |
| BMO area, *mm^2^* | 0.946 | (0.516, 1.736) | 0.858 |  |  |  |  |
| **Angular deviation of LC/BMO offset, *°*** | **1.094** | **(1.053, 1.138)** | **<0.001** |  | **1.190** | **(1.116, 1.269)** | **<0.001** |
| **Offset Index** | **1143.55** | **(0.026, 5.12e+07)** | **0.197** |  | **6369015** | **(84.236, 4.82e+11)** | **0.006** |
| Angular deviation of β-zone PPA, *°* | 0.986 | (0.960, 1.013) | 0.314 |  |  |  |  |

OR = odds ratio; CI = confidence interval; IOP = intraocular pressure; BMO = Bruch’s membrane opening; LC = lamina cribrosa; PPA = parapapillary atrophy

Statistically significant values (*P*<0.05) are shown in bold. ^*^Multivariable analysis is performed using same variables with Table 2.

**Supplemental Table 2.** Factors associated with angular deviation of LC/BMO offset in glaucomatous eyes

|  | Univariable analysis | | |  | Multivariable analysis^*^ | | |
| --- | --- | --- | --- | --- | --- | --- | --- |
|  | Coefficient | 95% CI | *P* |  | Coefficient | 95% CI | *P* |
| **Age*, years*** | **2.860** | **(2.110, 3.610)** | **<0.001** |  | **1.253** | **(0.455, 2.050)** | **0.002** |
| Female (vs. male sex) | 58.390 | (25.457, 91.323) | 0.001 |  | -0.553 | (-22.662, 21.555) | 0.961 |
| **Axial length, *mm*** | **-21.309** | **(-29.277, -13.342)** | **<0.001** |  | **-10.845** | **(-17.993, -3.698)** | **0.003** |
| IOP, *mmHg* | 0.284 | (-2.944, 3.512) | 0.863 |  |  |  |  |
| BMO area, *mm^2^* | -7.451 | (-16.240, 1.338) | 0.097 |  | -0.187 | (-5.618, 5.245) | 0.946 |
| **Angular deviation of OPP, *°*** | **-0.822** | **(-1.028, -0.616)** | **<0.001** |  | **-0.439** | **(-0.649, -0.230)** | **<0.001** |
| Angular deviation of β-zone PPA, *°* | -0.019 | (-0.130, 0.158) | 0.848 |  |  |  |  |

CI = confidence interval; IOP = intraocular pressure; LC = lamina cribrosa; BMO = Bruch’s membrane opening; OPP = outermost protruded point; PPA = parapapillary atrophy

Statistically significant values (*P*<0.05) are shown in bold. ^*^Variables with *P*<0.10 in the univariable analysis were included in the subsequent multivariable analysis.
